# Social information modifies the associations between forest fragmentation and the abundance of a passerine bird

**DOI:** 10.1038/s41598-023-48512-8

**Published:** 2023-12-04

**Authors:** Michał Bełcik, Magdalena Lidia Lenda, Sylwia Pustkowiak, Bartłomiej Woźniak, Piotr Skórka

**Affiliations:** 1 Institute of Nature Conservation, Polish Academy of Sciences, Mickiewicza 33, 31-120 Kraków, Poland; 2 Centre for Biodiversity and Conservation Science, University of Queensland, Brisbane, QLD 4072 Australia; 3 Population Ecology Lab, Institute of Environmental Biology, Faculty of Biology, Adam Mickiewicz University, Uniwersytetu Poznańskiego 6, 61-614 Poznań, Poland; 4 Department of Forest Zoology and Wildlife Management, Institute of Forest Sciences, Warsaw University of Life Sciences – SGGW, Nowoursynowska 159, 02-776 Warsaw, Poland

**Keywords:** Behavioural ecology, Ecosystem ecology, Forest ecology

## Abstract

Habitat loss and fragmentation are the main factors driving the occurrence and abundance of species in the landscape. However, the local occurrence and abundance of species may also depend on conspecific and heterospecific social information e.g. clues of animals’ presence or their voices. We investigated the impact of the interaction between different types of social information and forest fragmentation on the abundance of the song thrush, *Turdus philomelos*, in Central Europe. Three types of social information (attractive, repulsive, and mixed) and procedural control were broadcasted via loudspeakers in 150 forest patches that varied in size and isolation metrics. Repulsive social information (cues of presence of predator) decreased abundance of song thrush. Also, the repulsive social information changed the association between forest patch isolation, size and the abundance. Attractive social information (songs of the studied thrush) had no effect on song thrush abundance. However, the attractive social information reversed the positive correlation between habitat patch size and the abundance. Mixed social information (both repulsive and attractive) had no impact on the abundance nor interacted with habitat fragmentation. The observed effects mostly did not last to the next breeding season. Overall, our findings indicate that lands of fear and social attraction could modify the effect of habitat fragmentation on the species abundance but these effects probably are not long-lasting.

Habitat loss and fragmentation drive major ecological processes (e.g., dispersal, population viability, and species interactions) across the globe^1–3^. According to the classic concepts of island biogeography^4^ and metapopulation theory^4,5^, the isolation of habitat patches negatively affects the occurrence and abundance of species in a fragmented landscape. In these concepts, larger and less isolated habitat patches should have a greater chance of being occupied by a given species and harbor a greater population density than smaller and more isolated patches^5,6^.

The spatial configuration of habitat patches is not the only factor that influences the local occurrence and abundance of species^7^. In fact, habitat selection is non-random process^8^and population characteristics may also depend on heterospecific and conspecific interactions among individual animals^9,10^. Owing to the high heterogeneity of habitat patches^11^, individuals may need to gather information on the environment to correctly assess local habitat quality^12^. As information gathered by one individual regarding the structural features of the landscape may be misleading for making vital decisions, animals may use social information during their assessment of the local habitat quality and selection of their subsequent breeding site^13^.

The term social information may refer to the presence of other individuals, their behavior, the sounds they make, or other traces of their presence or activity^10,14–16^. Social information can be carried by other heterospecific or conspecific individuals^17^, and may trigger a variety of behavioral responses, depending on the source and the recipient^10,18^. Signs of presence of predators are an important aspect describing the landscape of fear, a mental map describing continuous spatial variation in an animal's perception of predation risk, including places that an animal avoids minimizing risk^19^. Signs of the presence of predators might affect how potential prey utilize the landscape^20^ and may deter prey species from settling in a given area^21–23^, thus it may be perceived as repulsive information. On the other hand, acoustic signals (e.g. passerine songs) of conspecifics and heterospecifics may be perceived as attractive information, because animals can use the presence of a successfully established individual as an indicator of the location of a high-quality habitat or vital resources^24,25^, therefore attracting individuals and increasing population density^15,16^. Both of those types of information could often coexist in the same habitat patch, creating a mixed social information. Although a recent study investigated the role of social information on patch occupancy by local populations^16,26^, very few studies have addressed relative role of social information derived from different sources on the abundance in local populations. In the real world, the existence of only one type of social information is rare, as different species create a network of direct and indirect interactions^27^. This differential value of social information may modify the distribution of animals and their abundance depending on whether different types of social information cues occur jointly or alone.

Social information may also change impact of environmental variables on the distribution and abundance of animals^16,27,28^. Different types of social information may alter the impact of habitat fragmentation by diminishing or augmenting the effects of patch area and isolation on species occurrence and abundance. For example, providing attractive social information in suitable but small habitat patches may lead to the disappearance of the patch area effect and increase patch occupancy and the local abundance of a species in these patches^16^. Information derived from heterospecifics could be both attractive and repulsive, and it may trigger a density-dependent effect during the territory establishment^29^. Social information on the presence of a predator should markedly diminish the abundance in habitat patches as prey species should respond very rapidly to cues that strongly negatively correlate with their fitness^30^. This effect should be especially strong in small habitat patches, where shelter from predators may be insufficient^31^ relative to that of large habitat patches.

The duration of the effects of social information in the environment may vary among species. To date, the possibility of a carryover effect of social information (e.g. lasting more than one breeding season after the information presence) affecting the distribution of species has been addressed in few studies, mainly with negative results^16,32^. The revealing existence of such effect would significantly broaden our understanding of the role played by social information in shaping species distribution and population density in fragmented landscapes.

The song thrush *Turdus philomelos* (L.) is a good species for investigating the impact of social information on the distribution of individuals in a fragmented landscape. Song thrush is a medium-sized passerine bird that has a very distinctive mating call, allowing it to easily detect and locate territories, thereby determining its abundance^33^. Song thrush breeds at relatively high densities in its respective habitat in Central Europe^34^, and has been demonstrated to display a high degree of breeding philopatry^35^.

In this large-scale experimental study, we investigated the impact of the interaction between different types of social information and forest fragmentation metrics on the abundance index of song thrush by testing the following hypotheses:

## Hypothesis 1

Different types of social information (i.e., attractive, repulsive and mixed) differentially impacts on song thrush abundance. Repulsive social information has a negative effect on the abundance of song thrushes. Attractive social information has a positive effect on the abundance of song thrush. Mixed social information has no effect on song thrush abundance. Such effect is maintained in the following year if the carry-over effect occurs.

## Hypothesis 2

By creating an acoustic space that differs in its potential impact on song thrush abundance, social information modifies the correlation between song thrush abundance index and habitat patch size as well as isolation of forest patches. Attractive social information decreases the positive association between habitat patch size and song thrush abundance, and weakens the negative association between isolation and the abundance. Repulsive social information increases the positive correlation between habitat patch size and song thrush abundance index, as well as strengthens the negative correlation between isolation and the abundance index. Mixed social information does not modify the correlation between habitat patch size, isolation and song thrush abundance index. These effects are maintained in the following year if the carry-over effect occurs.

## Results

The mean number of song thrush individuals observed at a given forest patch per survey was 2.83 (SE = 0.19, min = 0, max = 14) in 2017, 2.48 (SE = 0.15, min = 0, max = 14) in 2018, and 3.06 (SE = 0.19, min = 0, max = 10) in 2019. At least one thrush was observed in 133 forest patches in 2017, 130 in 2018, and 135 in 2019.

### Hypothesis 1: Effect of different types of social information on song thrush abundance

**Figure 1:**
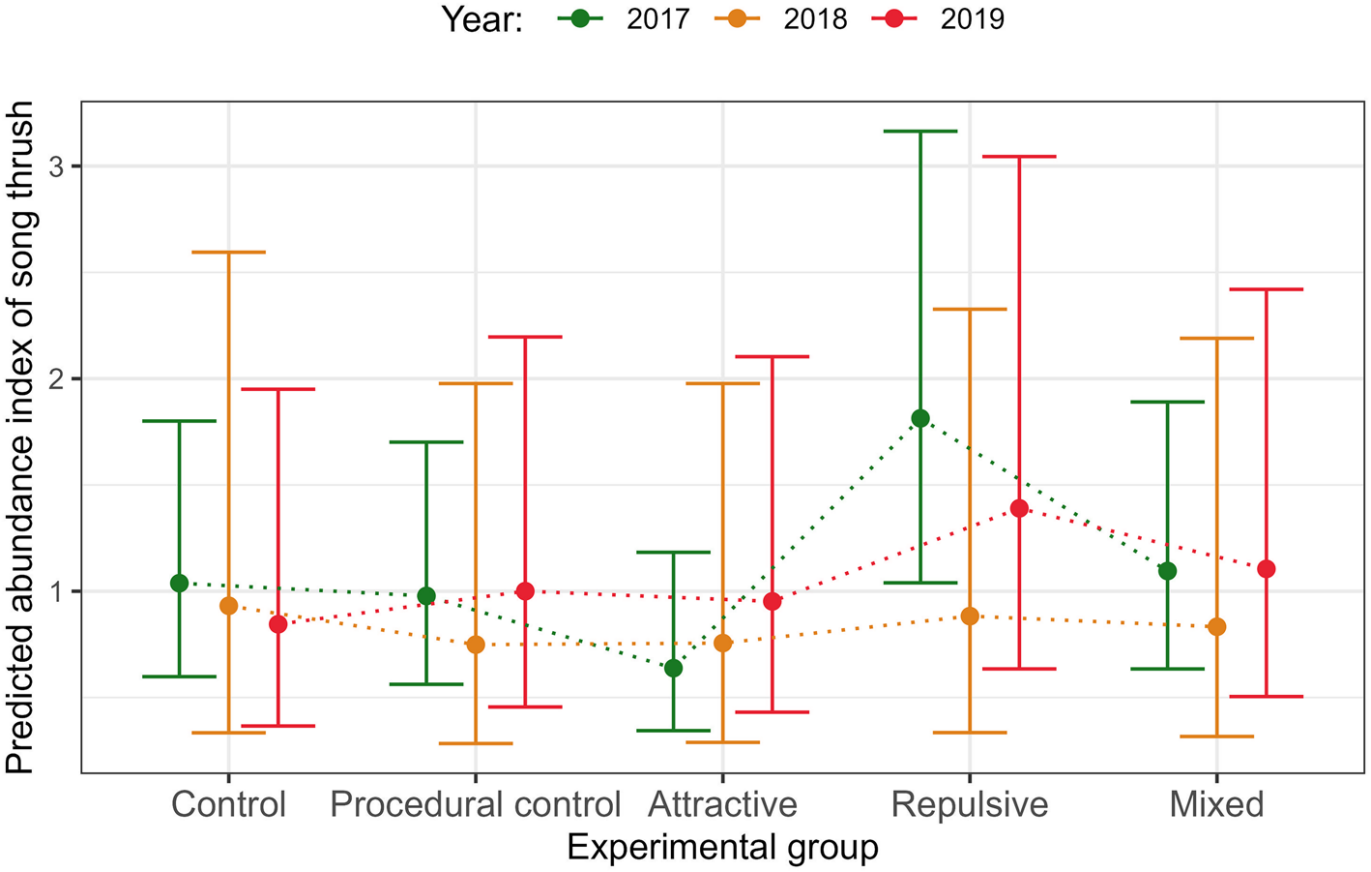
The estimated mean abundance index of song thrush (mean number of records per survey) per forest patch within different experimental groups during three years of the study. Whiskers are 95% confidence intervals. For significant differences see: Table 2.

Herein, before the experiment we noted statistically significant differences in the abundance index of song thrush in forest patches assigned to different experimental groups (Table 1). Namely, the abundance index in 2017 was lower in forest patches assigned to attractive social information broadcast (Tables 1 and 2, Fig. 1) and higher in patches assigned to repulsive social information broadcast (Tables 1 and 2, Fig. 1) as compared to patches assigned to control and procedural control forest patches. In years 2018 and 2019 there was no statistically significant response in abundance index among forest patches subjected to different social information types (Tables 1 and 2, Fig. 1). However, analysis of relative abundance (difference in abundance between 2017 and 2018) revealed that in forest patches where repulsive social information was broadcasted the abundance of song thrush decreased more in 2018 than in forest patches where control, procedural control and attractive social information was broadcasted (Tables 2 and 3). Moreover, the relative abundance index was statistically lower in forest patches with repulsive broadcast in 2019 as compared with patches with attractive social information (Table 2).

**Table 1:** The effect of the broadcasted information types and isolation metrics on song thrush abundance.

Explanatory variables	Song thrush abundance in 2017 (GAMM 1)	Song thrush abundance in 2018 (GAMM 2)	Song thrush abundance in 2019 (GAMM 3)
GAMM estimates of function slopes with standard errors (in brackets) for linear predictors			
Intercept	− 3.373 (0.245)***	− 4.295 (0.354) ***	− 3.498 (0.265)***
Broadcast: Procedural control	− 0.059 (0.139)	− 0.218 (0.213)	0.169 (0.194)
Broadcast: Attractive	− 0.722 (0.230)**	− 0.203 (0.212)	0.120 (0.194)
Broadcast: Repulsive	0.406 (0.129)**	− 0.053 (0.224)	0.311 (0.199)
Broadcast: Mixed	0.054 (0.134)	− 0.112 (0.219)	0.268 (0.198)
Survey: 2	0.059 (0.091)	− 0.312 (0.303)	0.181 (0.251)
Survey: 3	− 0.067 (0.077)	0.317 (0.124)*	0.072 (0.096)
Accipiter: sp1	− 0.139 (0.209)	0.008 (0.125)	− 0.113 (0.108)
Turdus: sp1	− 0.146 (0.235)	0.709 (0.349)*	0.167 (0.259)
Turdus: sp2	− 0.239 (0.262)	0.935 (0.359)**	0.352 (0.278)
Turdus: sp3	0.541 (0.654)	−	0.365 (0.662)
Number of loudspeakers	−	0.061(0.100)	− 0.117 (0.086)
Effective degrees of freedom are given, approximate significance of smooth terms			
Log(NNDist)	0.423	0.001	0.000
Log(Forest area)	1.742***	2.220**	2.217***
Log(NNDist) × Broadcast: Control	0.001	0.001	0.737*
Log(NNDist) × Broadcast: Procedural Control	0.000	0.839*	0.000
Log(NNDist) × Broadcast: Attractive	0.558	0.333	0.000
Log(NNDist) × Broadcast: Repulsive	0.000	0.001	2.110**
Log(NNDist) × Broadcast: Mixed	0.000	0.002	0.000
Log(Area) × Broadcast: Control	0.000	0.001	0.000
Log (Area) × Broadcast: Procedural Control	0.350	0.000	0.000
Log(Area) × Broadcast: Attractive	1.658***	0.429	0.000
Log(Area) × Broadcast: Repulsive	1.801***	0.000	0.000
Log(Area) × Broadcast: Mixed	0.000	0.702	0.000
X,Y	6.306***	3.708***	5.586***
Date (numeric)	0.000	2.102***	1.785*
Survey start time	0.925***	1.137*	2.699***
Temperature	2.509**	0.001	0.300
Cloudiness	0.000	0.000	0.000
Number of other bird species	0.000	0.000	0.000
Forest identity (random effect)	0.000	18.580*	16.850*
Deviance explained (%)	39.7	38.3	47.0

Estimates from generalized additive mixed models with Poisson error distribution. In every model survey duration (in minutes) was included as the offset variable. Statistically significant effects are emboldened.

*p < 0.05, **p < 0.01, ***p < 0.001.

**Table 2:** Results of the comparison between categorical predictor levels for song thrush abundance index.

Comparison	Estimate	SE	χ2	*df*	*P*
GAMM 1					
Control with Procedural control	−0.059	0.139	0.184	1	0.668
Control with Attractive	**−0.722**	**0.230**	**9.878**	**1**	**0.002**
Control with Repulsive	**0.406**	**0.129**	**9.917**	**1**	**0.002**
Control with Mixed	0.054	0.135	0.160	1	0.689
Procedural control with Attractive	**−0.662**	**0.231**	**8.253**	**1**	**0.004**
Procedural control with Repulsive	**0.466**	**0.132**	**12.519**	**1**	**0.000**
Procedural control with Mixed	0.113	0.138	0.676	1	0.411
Attractive with Repulsive	**1.128**	**0.224**	**25.400**	**1**	**0.000**
Attractive with Mixed	**0.776**	**0.227**	**11.719**	**1**	**0.001**
Repulsive with Mixed	**−0.353**	**0.123**	**8.195**	**1**	**0.004**
GAMM 2					
Control with Procedural control	−0.218	0.213	1.050	1	0.305
Control with Attractive	−0.204	0.212	0.926	1	0.336
Control with Repulsive	−0.053	0.224	0.057	1	0.811
Control with Mixed	−0.112	0.219	0.261	1	0.609
Procedural control with Attractive	0.014	0.153	0.009	1	0.926
Procedural control with Repulsive	0.165	0.143	1.321	1	0.250
Procedural control with Mixed	0.106	0.149	0.512	1	0.474
Attractive with Repulsive	0.150	0.153	0.961	1	0.327
Attractive with Mixed	0.092	0.148	0.385	1	0.535
Repulsive with Mixed	−0.058	0.143	0.166	1	0.684
GAMM 3					
Control with Procedural control	0.169	0.194	0.755	1	0.385
Control with Attractive	0.120	0.194	0.381	1	0.537
Control with Repulsive	0.311	0.199	2.453	1	0.117
Control with Mixed	0.268	0.198	1.829	1	0.176
Procedural control with Attractive	−0.049	0.131	0.141	1	0.707
Procedural control with Repulsive	0.142	0.120	1.395	1	0.238
Procedural control with Mixed	0.100	0.126	0.622	1	0.430
Attractive with Repulsive	0.191	0.131	2.150	1	0.143
Attractive with Mixed	0.149	0.131	1.298	1	0.255
Repulsive with Mixed	−0.043	0.123	0.121	1	0.728
GAMM 4					
Control with Procedural control	−0.077	0.332	0.053	1	0.817
Control with Attractive	0.111	0.328	0.114	1	0.736
Control with Repulsive	**−0.761**	**0.346**	**4.828**	**1**	**0.028**
Control with Mixed	−0.266	0.322	0.683	1	0.409
Procedural control with Attractive	0.187	0.275	0.465	1	0.495
Procedural control with Repulsive	**−0.685**	**0.273**	**6.290**	**1**	**0.012**
Procedural control with Mixed	−0.189	0.280	0.458	1	0.498
Attractive with Repulsive	**−0.872**	**0.278**	**9.824**	**1**	**0.002**
Attractive with Mixed	−0.377	0.282	1.784	1	0.182
Repulsive with Mixed	0.495	0.274	3.263	1	0.071
GAMM 5					
Control with Procedural control	−0.102	0.358	0.081	1	0.776
Control with Attractive	0.196	0.345	0.322	1	0.570
Control with Repulsive	−0.529	0.371	2.033	1	0.154
Control with Mixed	−0.209	0.345	0.366	1	0.545
Procedural control with Attractive	0.297	0.283	1.102	1	0.294
Procedural control with Repulsive	−0.428	0.281	2.319	1	0.128
Procedural control with Mixed	−0.107	0.281	0.145	1	0.704
Attractive with Repulsive	**−0.725**	**0.288**	**6.320**	**1**	**0.012**
Attractive with Mixed	−0.404	0.284	2.021	1	0.155
Repulsive with Mixed	0.321	0.284	1.277	1	0.258

Wald post−hoc test was used. Statistically significant comparisons are emboldened.

**Table 3:** The effect of the broadcasted information types and isolation metrics on song thrush relative abundance (difference in abundance between 2018 and 2017, and 2019 and 2017).

Explanatory variables		Song thrush relative abundance in 2018 (GAMM 4)	Song thrush relative abundance in 2019 (GAMM 5)
GAMM estimates of function slopes with standard errors (in brackets) for linear predictors			
Intercept		−4.796 (0.938)***	−3.669 (0.981)***
Broadcast: Procedural control		−0.077 (0.332)	−0.102 (0.358)
Broadcast: Attractive		0.111 (0.328)	0.195 (0.345)
Broadcast: Repulsive		−0.761 (0.346)*	−0.529 (0.371)
Broadcast: Mixed		−0.266 (0.322)	−0.208 (0.345)
Survey: 2		−1.398 (0.717)	−0.047 (0.568)
Survey: 3		0.359 (0.308)	0.369 (0.256)
Mean Accipiter: sp0.5		0.209 (0.245)	−0.285 (0.250)
Mean Accipiter: sp1		1.429 (0.815)	−0.247 (0.950)
Mean Turdus: sp0.5		−0.180 (0.935)	−0.360 (0.999)
Mean Turdus: sp1		0.524 (0.934)	−0.002 (0.963)
Mean Turdus: sp1.5		0.843 (0.961)	0.249 (0.999)
Mean Turdus: sp2		0.841 (1.141)	1.477 (1.141)
Number of loudspeakers		0.118 (0.123)	−0.001 (0.141)
Effective degrees of freedom are given, approximate significance of smooth terms			
log(NNDist)		0.001	0.001
log(Forest area)		0.001	0.502
log(NNDist) × Broadcast: Control		0.000	1.192*
log(NNDist) × Broadcast: Procedural Control		1.540***	0.398
log(NNDist) × Broadcast: Attractive		0.000	0.000
log(NNDist) × Broadcast: Repulsive		0.000	0.000
log(NNDist) × Broadcast: Mixed		0.165	0.000
log(Area) × Broadcast: Control		0.001	0.001
log (Area) × Broadcast: Procedural Control		0.660	0.000
log(Area) × Broadcast: Attractive		1.350*	0.077
log(Area) × Broadcast: Repulsive		0.000	0.102
log(Area) × Broadcast: Mixed		0.000	0.000
X,Y		1.939	0.000
Mean date (numeric)		2.007**	0.820
Mean Survey start time		0.001	0.000
Mean Temperature		0.522	0.921***
Mean Cloudiness		1.485*	0.524
Mean Number of other bird species		3.616***	0.929*
Forest identity (random effect)		3.495	0.004
**Deviance explained (%)**		27.7	13.3

Estimates from generalized additive mixed models with Gaussian error distribution. In every model total survey duration (in minutes) was included as the offset variable. Statistically significant effects are emboldened.

*p < 0.05, **p < 0.01, *** *p* < 0.001.

### Hypothesis 2: Modification of the effect of habitat fragmentation on song thrush abundance index

Overall, there were no significant correlations between forest isolation index and the abundance index (Table 1). There were statistically significant positive correlations between forest patch size and abundance index of song thrush (Table 1, Fig. S1 in Supporting information). However, there were significant interactions between forest fragmentation metrics and social information broadcast type (Tables 1, 2 and 3).

**Figure 2:**
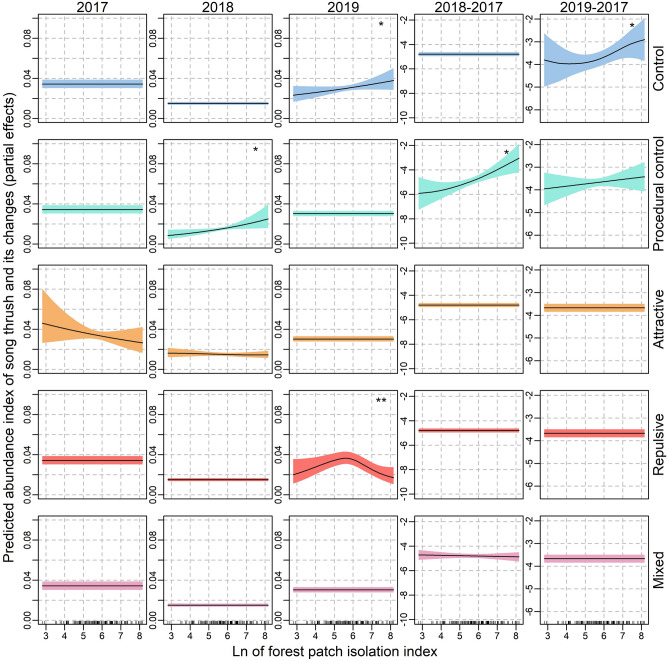
The interactive effects of forest patch isolation and experimental procedure on the abundance index of song thrush (columns 1–3) and relative abundance index (difference in the abundance between year 2018 and 2017—column 4; and between 2019 and 2017—column 5). Partial estimates from the generalized additive mixed models with Poisson error distribution (columns 1–3) or Gaussian error distribution (columns 4–5). In every model survey duration was included as the offset variable. Ribbons indicate 95% confidence intervals of the estimate. Explanations: *p < 0.05, **p < 0.01.

There were no statistically significant correlations between the song thrush abundance index and the forest isolation index in any forest groups assigned to experimental treatments in 2017 (Table 1; Fig. 2). However, in 2018 the positive correlations were found in forest patches where procedural control was broadcasted and in 2019 in control forest patches (Table 1; Fig. 2). These correlations were also visible in the relative abundance index (Table 3; Fig. 2). Moreover, a non-linear association between forest isolation index and the abundance index of song thrush in forest patches where repulsive information was broadcasted emerged in 2019—the abundance index peaked at moderate values of forest isolation (Table 1; Fig. 2).

**Figure 3:**
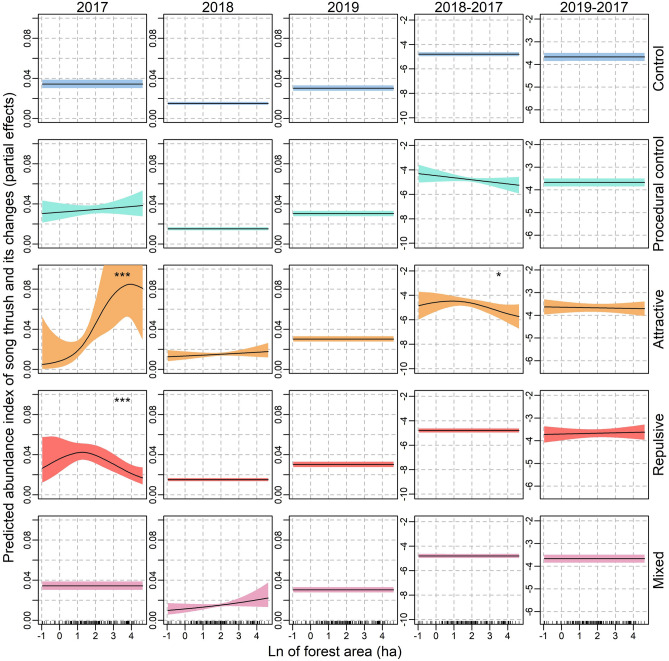
The interactive effects of forest patch area (natural logarithm) and experimental treatment on the abundance index of song thrush (columns 1–3) and relative abundance index (difference in the abundance between year 2018 and 2017—column 4; and between 2019 and 2017 column 5). Partial estimates from the generalized additive mixed models with Poisson error distribution (columns 1–3) or Gaussian error distribution (columns 4–5). In every model survey duration was included as the offset variable. Ribbons indicate 95% confidence intervals of the estimate. Explanations: *p < 0.05, ***p < 0.01.

There were statistically significant non-linear associations between song thrush abundance index and forest patch area in forest groups assigned to attractive (positive correlation) and repulsive (peak of abundance at moderate values of patch area) treatments in 2017 (Table 1; Fig. 3). However, after the experiment performed in 2018 these associations disappeared (Table 1; Fig. 3). This was partially confirmed by GAMMs for relative abundance index (Table 3; Fig. 3). Namely, the relative abundance (difference in abundance index between 2018 and 2017) decreased with forest patch area in forests with attractive treatment indicating that attractive social information led to decrease of abundance of song thrush in the largest forests and the increase in small ones (GAMM 4; Table 3; Fig. 3).

### Impact of other confounding variables: biotic interactions, weather conditions and survey characteristics

We found that in 2018 the number of other thrush species was positively associated with abundance index of the song thrush (Table 1). The number of other bird species did not correlate with abundance index of song thrush in any year (Table 1). However, the relative abundance index correlated negatively and non-linearly with the mean number of other species (Table 3, Fig. S2) indicating that the number of song thrushes decreased the most in habitat patches with large number of other species. We did not find the effect of already present potential predator species (*Accipiter* sp.) on the abundance index of song thrush and relative abundance index (Tables 1 and 3).

The abundance index of song thrush was also correlated with weather conditions in some years. Temperature was positively correlated with number of recorded song thrushes in 2017 (Table 1, Fig. S3) but not in other years. Moreover, mean temperature was negatively correlated with the relative abundance of song thrush (the difference in abundance index between 2019 and 2017; Table 3, Fig. S3) indicating that when temperatures were high during surveys the number of recorded song thrushes in 2019, as compared to 2017, decreased more than when mean temperatures were low. Mean cloudiness was positively associated with the relative abundance of song thrush (the difference in abundance index between 2018 and 2017; Table 3, Fig. S4) indicating that number of song thrushes decreased in 2018 as compared to 2017 especially when cloudiness was low (Table 3, Fig. S4).

We found that the abundance index of the song thrush decreased with time from the sunrise in each year but it also increased around the noon in 2019 (Table 1, Fig. S5). The date also had an effect on the abundance index of song thrush in 2018 and 2019 (Table 1). The abundance index was the highest at the beginning of April and late in May (Fig. S6). Also, the relative abundance index (the difference between the abundance index in 2018 and 2017) was non-linearly correlated with mean date (GAMM 4 in Table 3; Fig. S7) indicating that in surveys performed generally early in a season the number of recorded song thrushes in 2018, as compared with 2017, decreased the most.

## Discussion

In this study, deciding on a settling area was demonstrated to be a complex process for song thrush. Social landscape is one of the factors considered by this species, and may influence other factors, such as the structural characteristics of a habitat patch. Among the social information types, land of fear and social attraction are recognized to be the most significant, surpassing land of uncertainty.

### Response to a repulsive social information

We found that Northern goshawks may play a role in shaping the spatial distribution and abundance of song thrush. Indeed, abundance index of song thrush decreased in forest patches where territorial voices of the goshawk were broadcasted. It is an expected result because song thrush is a major component of the goshawk’s diet. According to previous studies, the abundance of potential prey species in large forest patches increases with increasing distance from goshawk nests^36^. However, whether the same phenomenon can be observed in fragmented forests in an agricultural landscape is unclear. A non-linear association was found between the forest isolation and song thrush abundance index in patches where repulsive social information was broadcasted. The abundance was the highest in forests moderately isolated. This result might be due to optimal habitat choice balancing between cost-efficiency to cross the landscape and higher probability of predation in both well connected and highly isolated forest patches^37,38^. According to previous research, goshawks do not hunt in the vicinity of their nests^39,40^. The size of the goshawk territory varies from 300 to 1000 ha in the study region^41^. Therefore, in small and isolated forest patches, a goshawk could forage outside of these patches, thereby decreasing predation risk and increasing patch attractiveness as a nesting location for a song thrush. In more isolated habitat patches, goshawks could also catch more mammals, including hares, than birds^42^. In larger and well-connected habitat patches goshawks may be more specialist hunter preferring some species (e.g. song thrush).

Goshawk is regarded as a forest species^36,43^, and in Poland, this species nests in lower density in agricultural landscapes. The population of goshawks inhabiting agricultural landscapes in Poland is declining^44,45^. Occupancy models suggest that the decline in a species population may start with the species leaving the suboptimal habitat^46^. Relatively small forests spread in agricultural landscape may be therefore suboptimal habitat for goshawk. Even in continuous forest habitat, goshawks can abandon areas where the share of clear cuttings exceeds 30% of their territory^47^. Thus it is possible that in some forests goshawk may not be perceived by song thrush as major predatory threat.

### Response to an attractive social information

Despite we did not find the simple effect of attractive social information on the abundance index of studied species there was change from the positive correlation in 2017 to no correlation between forest patch area and song thrush abundance index in forests with this information type. This change is in line with our expectations. Since attractive social information could be generally perceived as a sign of a high-value habitat, the observed changes would suggest that clues of such habitat might be one of the factors influencing bird’s decision to settle as it is predicted by interspecific attraction hypothesis^48^. Additionally, our findings suggest that social cues may contribute to the reduction of perceived risk when an individual bird chooses to inhabit small habitat patches,

Additionally, our findings suggest that social cues may contribute to the reduction of perceived risk when an individual birds chooses to inhabit small habitat patches^18^, which are frequently regarded as suboptimal habitat.

### Response to the mixed social information

In our study, land of uncertainty (formed by mixed social information) had no effect on thrush abundance nor modified associations between forest patch size and isolation, and the abundance index. As mixed information is a combination of attractive information and repulsive information, one may suggest that song thrush might be deterred by the repulsive component of the mixed social information because it may have stronger impact on individual fitness. However, it was not the case in our study. This may indicate that different sources of social information may create real land of uncertainty, where other clues such as the structural components of a habitat patch or surrounding landscape may provide more valuable information on the habitat suitability and quality. Also, in multi-species communities individuals are entangled in many inter-specific interactions, many based on social clues. Consequently, it is possible that these clues constitute too many sources of social information varying in values and, eventually, they can not be correctly assessed, valued and used by animals. It is topic worth exploring in future studies as most of authors consider usually one social information type in their research.

### Effects observed in control and procedural control forest patches

Interestingly, we found that positive association between isolation and song thrush abundance index appeared in patches subjected to procedural control in 2018 and control ones in 2019 as compared to no effect in other years and most of treatments. There could occur some spatial shifts in distribution in song thrush territories due to experimental manipulation. The natal dispersal and breeding dispersal of this species is about 7 km and up to 20 km, respectively^49,50^, thus it is possible that individuals from forests where repulsive social information was broadcasted moved or colonized forest patches where social clues were not provided (thus there were no repulsive signs of predators nor were other song thrush territories overcrowded). One of the ways to validate such hypothesis would be marking birds (by GPS receivers—see research limitations below) and tracking their movements among forest patches subjected to experimental addition of different social information types.

### Existence of the carryover effect

We did not find evidences for long-lasting effects of social information in song thrush local populations. This is an unexpected result especially in case of repulsive social information. Former research suggested that bird community structure near goshawks remains altered even a few years after the territory abandonment by the predator^36^. One possible explanation is high breeding philopatry^50^ that may reduce the effect of social information on decision-making for where to settle. However, the breeding philopatry has large variation and it is not equal among individuals because many birds settle in new sites. For example, estimates of natal dispersal for Song thrush is about 7 km on average with large variation^49^ and over 90% of Song thrushes disperse to within the distance of 20 km from breeding place (only less than 3% had moved to distant breeding sites)^50^. It is also possible that environment varies in time (e.g. food resources) thus song thrushes must assess breeding habitat before every breeding season. Moreover, life-span of song thrush is not long (~ 3 years) thus every year some new, unexperienced recruits appear in the area, also needing to assess the habitat suitability. Therefore, natal and breeding philopatry, varying environment and turnover of individuals may lead to dilution of the social information in time.

### The impact of other confounding variables

We found that some of the variables associated with biotic interactions, weather conditions and survey characteristics played a role in estimation of song thrush abundance. Contrary to expectations, number of other thrush species (*Turdus* sp.) had positive association with song thrush abundance index. It is possible that other thrushes prefer similar habitat characteristics (at least partially) which leads to positive co-occurrence. Also, song thrush may be a social facilitator, a species indicating safe and high-quality habitat and thus attracting other species.

Presence of potential predators (*Accipiter gentilis* and *A. nisus*) did not correlate with the abundance index of song thrush. It seems to contradict our findings where repulsive social information lowered abundance index of song thrush. However, the predators were detected only during 4–11% of surveys indicating that these predators are uncommon in the area. Also, the absence of the effect of potential predators on the song thrush abundance index may be explained by the fact that goshawk and sparrowhawk territories remain largely unchanged from year to year and that breeding pairs use the same nests for an extended period of time. As a result, song thrushes may be familiar with the area that the predator has already occupied. Other studies have found that the composition of local bird populations and communities might be conditional on past species interactions with *Accipiter gentilis*^36^.

Although the number of other bird species did not have an impact on the abundance of song thrushes in any one year, there was a substantial inverse association between the relative abundance index of song thrushes and the number of species. This finding suggests that forest patches with the greatest diversity of other bird species experienced the greatest changes (decreases) in the abundance index between years. Thus, it might imply that interactions between the song thrush and other potentially competing species affect how common song thrush is.

Weather conditions and survey time had some effect on the abundance of song thrush in 2017 but not in other years. The number of recorded individuals was positively correlated with temperature which may be associated with singing activity. Birds usually have higher singing rate and duration at higher temperatures^51^. Number of recorded song thrushes was the highest early in the morning. This is well known phenomenon for this and other bird species^49^. However, in 2019 we also observed increased number of records of song thrush at the noon. This could have been an effect of both increased singing and foraging activity. Moreover, number of song thrush records increased both at the beginning April and end of May in 2018 and 2019. This corresponds well with seasonal pattern of vocal activity of this species^52^. In conclusion, it is evident that weather during the survey and the time it is conducted have a significant impact on the estimates of abundance and should be controlled in similar research.

### Study limitations

In our experimental design, we did not account for social information that already existed in any of the studied forest patches, which serves as the main limitation of this study. Song thrush and goshawk had already resided in the area prior to our study. We controlled the effect of the predator presence in our statistical models. However, full control of the already present social information (e.g., capturing and removing birds from forest patches) was beyond the available means and raised serious ethical issues. Therefore, all conclusions drawn from this study can only be interpreted as the social information added to a given forest patch during the experiment.

Herein, we did not account for the detectability of song thrush in our analyses, thereby serving as another limitation of our study. However, we partially addressed this issue by controlling survey characteristics (temperature, cloudiness, surveys duration, survey starting time, date) that could be associated with the song thrush detectability in our statistical models. We also argue that species detection probability is not major concern given high vocal activity of this species. We detected song thrush in most of our forest patches thus we had to resign from analysis of forest occupancy. Moreover, our estimates of song thrush abundance correspond well with independent results collected via Monitoring of Birds of Poland^53 xxx^. Overall, the total population size of the song thrush in Poland shows increasing trend with certain year-to-year variation. In the year 2017, the abundance index in Poland attained its highest recorded value in the monitoring. Conversely, in 2018, the index reached its lowest value for the second decade of the twenty-first century. Therefore, our abundance index align closely with the country-level estimates.

We chose to count song thrush instead other potentially suitable methods. For, example capture-mark-recapture (CMR) is supposed to give very accurate estimates of population sizes^54^. However, CMR has also several disadvantages that make this method difficult for implementation in research on such large scale. There are also serious ethical issues in using this method^55^. CMR requires to capture and uniquely mark animals^54,56^. The repetitive capture and tagging procedures may not align with conservation strategies for protected and vulnerable species and is highly expensive^57^. Furthermore, acquiring a sufficiently large sample of individuals through trapping and marking can be challenging, and it may influence animal behavior, thereby introducing biases in population size estimates, especially in behavioural experiments. Consequently, there is a preference for non-invasive methods of population size estimation that do not rely on trapping^58^. More importantly, CMR in not ideal method to estimate population size for territorial birds^59^. Most of CMR methods assume certain level of “mixing” of individuals within a population which is, of course, not true in territorial animals, such as song thrush.

Also, passive acoustic recording^60^ could have been used to sample song thrush vocalizations and infer their behavioural response to acoustic environment as well as background noise. Passive acoustic recording is an excellent methodology but can be applied on a fine spatial scale because recordings in many forest patches would require enormous financial and human resources. Moreover, with this method it is almost impossible to control the effect of recorders on the behavior of birds and estimates of their abundance (no pure control treatment).

Further, we did not control man-made alteration in the environment. Although many forest patch characteristics (patch size and isolation metrics) were constant in time, we did not monitor for example some changes in forest management. Any clearcuts that could influence the abundance and spatial distribution of song thrushes within a forest patch were not mapped. However, clear-cuts alter the median age of the trees within the forest patch by introducing areas where the median age drops to zero (or to a single digit value, after a new tree generation has already been introduced). Therefore, the issue of clear-cuts and commercial felling was addressed by accounting for the median age of the trees in the main forest story, weighted by the area occupied by each of the similar-aged tree groups. For this experiment, forest patch groups were selected to ensure no difference in the weighted median age between the groups.

## Methods

### Study system

In this study, we considered three types of social information. The repulsive social information that created the land of fear was the territorial calls of the northern goshawk *Accipiter gentilis* (L.)*.* This large sized (male body length and mass are 46–61 cm and 360–1000 g, respectively; females are 59–70 cm in length and 770–2200 g in weight) top predator preys on different birds^36,61,62^and breeds in various forests and even in small patches. Therefore, presenting cues for the presence of this predator may deter many birds from settling in territories in forest habitat patches^63^. Northern goshawk vocalizes early during the breeding season, usually in March.

Attractive social information comprised songs of the song thrush. Song thrush is a medium-sized (20–23 cm in length and weighs 50–100 g) forest specialist species that occurs in different forest types. In Central Europe, song thrush is mainly a migratory bird that starts singing and settling in territories in March and chick rearing season peaking in May. Its songs are loud and can be heard from a distance, even outside the forest habitat patch^64^. The song thrush forages different food sources, both invertebrates and plants (e.g., fruits)^62^, and is one of the main prey species of northern goshawk^61^. Thus, the presence of song thrush may be a good cue of a good habitat type that is rich in food resources and free of predators.

Mixed social information, which creates a land of uncertainty, is the voice of both the northern goshawk and song thrush emitted alternately.

Our research was carried out from 2017 to 2019, and mainly consisted of two tasks. The first task involved the performance of field surveys on the abundance of song thrush in all selected forest patches, and the second involved a behavioral landscape experiment in which social information was manipulated in selected forest patches.

### Study sites

A study area in the southern part of Poland, in the province of Lesser Poland, north of Cracow, covering an area of 1,097 square kilometers, was selected. A total of 150 forest patches located in an agricultural landscape were surveyed. These patches were mainly mixed stands managed by the Polish State Forests Holding and private entities (supervised by the former entity). Of note, most of these forest patches are not part of a larger continuous forest complex and differ in size and isolation. The characteristics of the forest patch size were (in hectares) as follows: mean = 14.91, SD = 19.15, range 0.38–102.28. The forest patch isolation (Nearest Neighbour Distance) characteristics were (as meters): mean = 557.67, SD = 772.07, range 16.53–3509.19. These characteristics did not differ among experimental groups (Tables S1, S2 and S3 in Supporting information).

### Field surveys

Field surveys to estimate song thrush abundance were conducted between April 1 and June 1 in 2017–2019. Eleven surveys were done also between 2 and 7 June 2019 due to unfavorable weather conditions at the end of May. The surveys were conducted by a team of three experienced birdwatchers. However, two additional observers surveyed forest patches in 2017 due to health problems of formerly designated observer. Each observer had an assigned set of forest patches, with each patch being visited three times, once during each 20-day round (April 1–20, April 21–May 10, May 11–31). Surveys began at approximately 5 AM and usually lasted until noon (in April) or 11 AM (in May). One observer surveyed 4.4 ± 1.4 (mean ± SD) patches per day on average with range from 1 to 9 patches. During the surveys, an observer recorded the starting time and then moved through the forest in a random direction, attempting to cover as much of the forest patch as possible. Start times at sites were rotated between surveys. The rotation was done so that second survey was usually performed in opposite order to first survey. The third survey was performed starting from the middle set of forest patches. Thus, average starting time of the survey was similar among forest patches and different experimental groups. The observer recorded the exact time of every individual song thrush heard or seen within a patch and marked its location on a handheld GPS device. The ending time of the survey was also noted in every patch.

### Experimental manipulation in social information

The field experiment was conducted from March 17 to March 30, 2018, just before the breeding period of the song thrush, and before the start of the surveys. Before the experiment was initiated, the forest patches were assigned to one of five groups. Groups were selected so none of them differed statistically from other groups in terms of forest stand characteristics. The groups were as follows:

- Group of 30 patches where song thrush (*Turdus philomelos*) songs were broadcasted, which constituted attractive social information;
- Group of 30 patches where goshawk calls were broadcasted, which constituted repulsive social information;
- Group of 30 patches where both song thrush and goshawk calls were broadcasted, which constituted mixed social information;
- Group of 30 patches where background noise (i.e., sound of moving trees, wind, and sounds from the surrounding landscape) were broadcasted, serving as a procedural control;
- Group of 30 patches with no broadcast, serving as the control.

Within each group, a specific type of broadcast was assigned and played daily from 7 AM to 12 PM throughout the entire duration of the experiment. . The number of loudspeakers depended on the forest patch area, varying from a single loudspeaker to five loudspeakers. The number of loudspeakers was proportional to the forest size and it was selected to ensure that the entirety of each of the forest patch was covered with the range of broadcast of the loudspeakers, giving a total number of 177 speakers being operated daily. After the broadcast ended for a given day, the speakers were collected, charged, and hung the next morning.

Each broadcast had the same general pattern and consisted of five minutes of songs/calls and fifteen minutes of silence, repeated alternately for five hours. The broadcast of attractive social information was composed of one hour-long fragment, repeated five times to get five hours of broadcast in total. Each five-minutes broadcast was built of song exemplar from one male. The three exemplars from three different individuals were used for the whole playback. The broadcast of repulsive social information was built in similar way, but here, exemplars of calls of four individuals were used to build basic one-hour playback, that was repeated to obtain five hours of broadcast. The broadcast of mixed social information was composed from exemplars of attractive and repulsive information arranged alternately and intersected with 15 min of silence, which gave a two-hour fragment repeated subsequently to obtain five-hour long playback (Fig. S8). The procedural playback was constructed similarly, but contained a neutral ambient sound instead of songs/calls.

Bird song/voice recordings were obtained from the Xeno Canto portal, a website created for sharing bird voices from around the world (https://www.xeno-canto.org/). Only recordings that had an “A” mark for quality (indicating the highest quality available for this portal, no other species singing in the background and minimal noise) were used. A JAM HX-P710 speaker (set for maximum loudness, 80 dB) paired with a Philips GoGear Azure SA5AZU08KF mp4 player was employed as the broadcasting device.

### Data analysis

#### Fragmentation metrics

We calculated the habitat patch size and habitat patch isolation. Patch size was calculated in hectares. To measure isolation, two metrics that measure edge-to-edge isolation were computed: the nearest neighbor distance (NNDist) and proximity index (PROX). These metrics were found to be significantly correlated (r = -0.203, p < 0.01); however, NNDist was selected for the analysis as it had less skewed distribution and no outliers after log transformation, in contrast to PROX. All fragmentation metrics were calculated using the Patch Analyst toolbox in ArcGis ver. 10.1.

#### Statistical models

All statistical analyses were performed using R statistical software^65^. We constructed a series of models that would explain whether social information, fragmentation metrics, or interaction between the social information type and fragmentation metrics influenced (1) the abundance index and (2) the relative abundance of song thrush in each of the studied forest patches. The abundance index was the number of song thrush records during each survey in a given forest patch. The relative abundance was the difference in the abundance index between 2017 and 2018, and between 2017 and 2019 (difference in abundance index between 1st survey in 2018 or 2019 and 2017, and so on). Statistical models were constructed using the “mgcv” package^66^. A generalized additive mixed model was constructed for each of the response variables: abundance index in 2017 (GAMM 1), in 2018 (GAMM 2), in 2019 (GAMM 3) and relative abundance in 2018 (GAMM 4) and 2019 (GAMM 5). The models included all explanatory variables that might explain the song thrush abundance variability: social information type used during the experiment (attractive, repulsive, mixed, procedural control and control), fragmentation metrics (NNDist, patch area), and factor-smooth interactions between each information type and fragmentation metric. Moreover, in each GAMM we included following covariates that may affect abundance and detectability of song thrush:

- Number of other bird species noted during the survey (numeric variable),
- Number of other thrush species as the estimation of the number of potential competing species. The variable was expressed as factor (levels: 0—none, 1—one species, 2—two species, 3—three species),
- Number of potential avian predators which was the number of *Accipiter sp.* The variable was expressed as a factor (levels: 0—none predator detected, 1—one species, 2—two species),
- Survey (factor with three levels: first, second, third),
- Date (numeric variable expressed as the number of days from 1^st^ April),
- Survey duration, measured in minutes. This variable (transformed via natural logarithm) was used as the offset in GAMMs. Thus, GAMMs results should be interpreted in the scale of this variable, e.g. number of song thrush records per minute,
- Survey start time, measured as the number of minutes from the sunrise at a given site and date. The variable was calculated with help of function ‘sunrise()’ from ‘bioRad’ package^64^,
- Temperature during a survey (in degrees of Celsius),
- Cloudiness during a survey measured visually in cloud cover categories at the beginning of survey (0, 1–10%, 11–20%,…90–100%),
- Number of loudspeakers (for GAMMs 2–5).

For GAMMs 4 and 5 that were based on the differences in number of song thrush records, we used mean values of the above listed variables for each survey (e.g. in GAMM 4 the mean value of date for survey one in 2017 and 2018 was calculated in each forest patch), except survey duration that was sum of time for consecutive surveys in two years (e.g. total time spent in a given forest during survey one in 2017 and 2019). As our analyses were based on survey-level data we included also forest patch identity as the random factor via specifying smooth parameter bs = “re” as recommended in Wood^66^. Moreover, the interaction between geographic coordinates modeled as a smoothed function for all models was also included to account for spatial autocorrelation. The fragmentation metrics were transformed by natural logarithm to avoid the impact of outlying data points and a non-linear relationship was assumed for all explanatory continuous numeric variables, except variables coded as factors (e.g. information type). To check for adequacy of our models, the ‘model.check()’ function from the “mgcv” package was used, which produces diagnostic information and residual plots. The ‘concurvity()’ function from the same package, which produces summary measures of the concurvity between model components, was also employed. These checks collectively revealed correct construction of the statistical model (all values of observed concurvity lower than 0.8). A post-hoc Wald test was subsequently performed to check the differences in the abundance index or relative abundance responses to each of the social information types, using the ‘wald_gam()’ function from the “itsadug” package^67^.

To test our first hypothesis, we checked the statistical significance of the social information-type variable and the Wald test results. To test our second hypothesis, we checked the statistical significance of interactions between information type and fragmentation metrics and inspected plots showing the relationship between abundance index in 2017 (GAMM 1) and 2018 (GAMM 2) as well as the difference in abundance index between years 2017 and 2018 (GAMM 4), and fragmentation metrics at a given type of social information. To test for carryover effects, we have checked whether the effect of the experiment was statistically significant in the year 2019 (GAMM 3) and the difference in abundance index between 2017 and 2019 (GAMM 5). In GAMMs visualization of interaction terms we added the intercept by specifying the argument ‘shift = TRUE’ and adding the uncertainty associated with mean estimates ‘seWithMean = TRUE’ in ‘mgcv’ R package.

### Supplementary Information

Supplementary Information.

## Data Availability

All data are attached to the manuscript and will be made available upon request to authors.
