# Supplementary material for: Social information modifies the associations between forest fragmentation and the abundance of a passerine bird
Source: Sci Rep. 2023 Dec 4;13:21386. doi: 10.1038/s41598-023-48512-8 (PMC10696010; doi:10.1038/s41598-023-48512-8)
Supplement: Supplementary file 1 — Supplementary Information. [file 41598_2023_48512_MOESM1_ESM.pdf]

Bełcik M., Lenda M.L., Pustkowiak S., Woźniak B., Skórka P.

Supplementary information

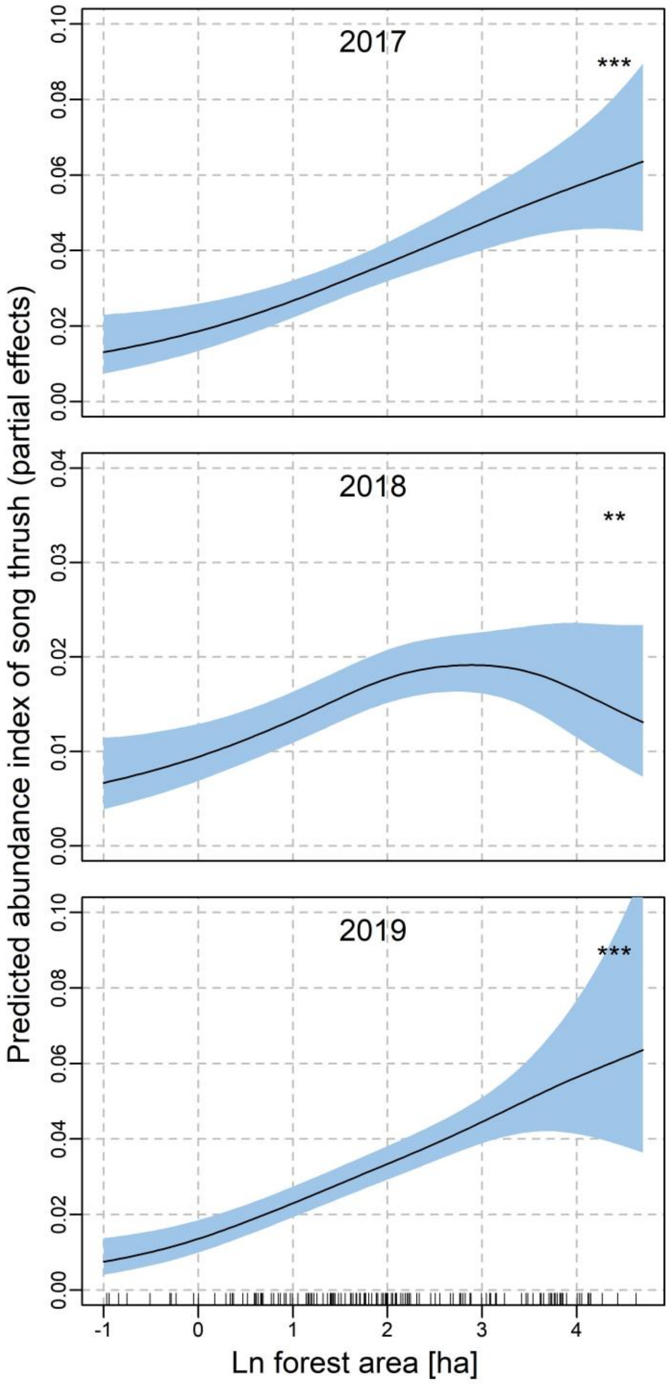

Figure S1. The association between the forest patch size (transformed by the natural logarithm) and abundance index of song thrush. Estimates from generalized additive mixed models with Poisson error distribution. In every model survey duration (in minutes) was included as the offset variable. Ribbons are 95 % confidence intervals. Explanations: ** - $p < 0.01$, *** - $p < 0.001$.

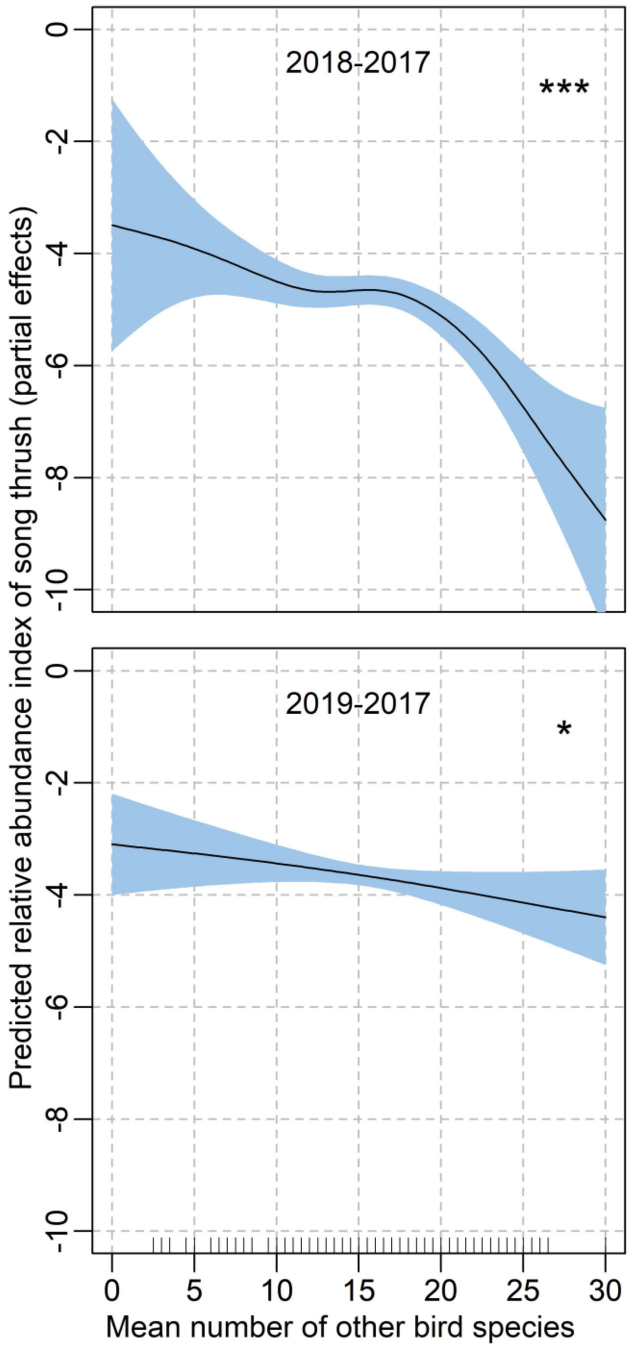

Figure S2. Effect of the mean number of other bird species (excluding song thrush) on the relative abundance index of song thrushes (differences in abundance index between 2018 and 2017, and between 2019 and 2017). Estimates from generalized additive mixed models with Gaussian error distribution. In every model total survey duration (in minutes) was included as the offset variable. Ribbons are 95 % confidence intervals. Explanations: * - $p < 0.05$, *** - $p < 0.001$.

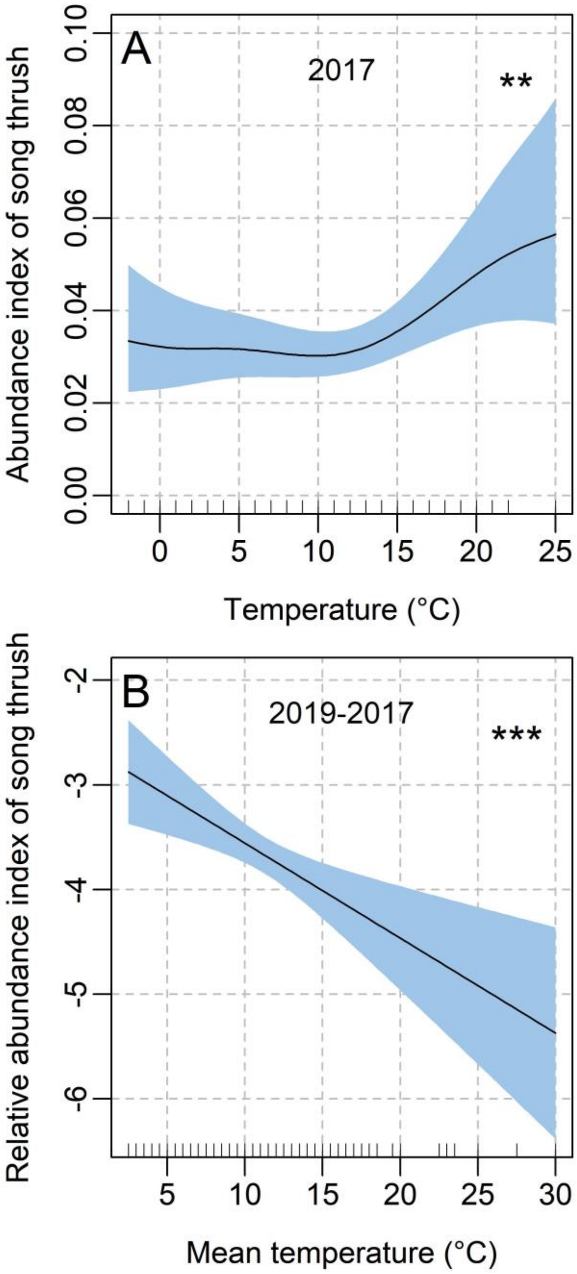

Figure S3. Effect of temperature (A) and mean temperature (B) on the predicted abundance index of song thrushes in 2017 and relative abundance index (2019-2017), respectively. Estimates from generalized additive mixed models with Poisson (A) and Gaussian (B) error distribution. In every model survey duration (in minutes) was included as the offset variable. Ribbons are 95 % confidence intervals. Explanations: ** - $p < 0.01$, *** - $p < 0.001$.

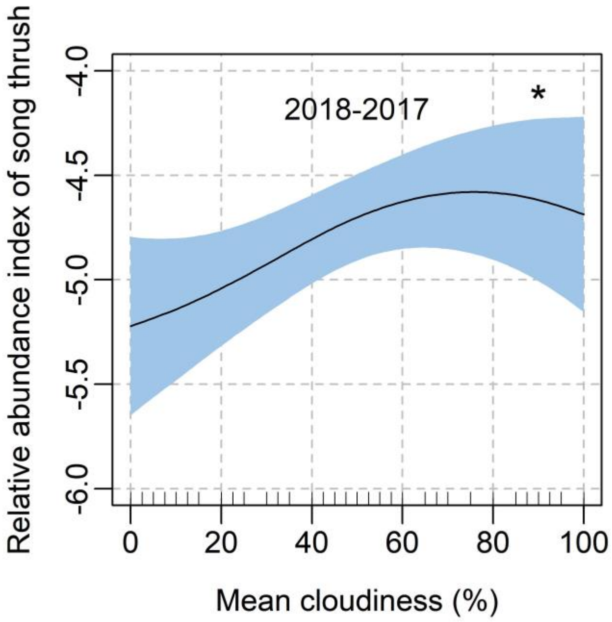

Figure S4. Effect of mean cloudiness on the relative abundance index of song thrush (difference in abundance index between 2019 and 2017). Estimates from the generalized additive mixed model with Gaussian error distribution. Total survey duration (in minutes) was included as the offset variable. Ribbons are 95 % confidence intervals. Explanations: * - $p <$ 0.05.

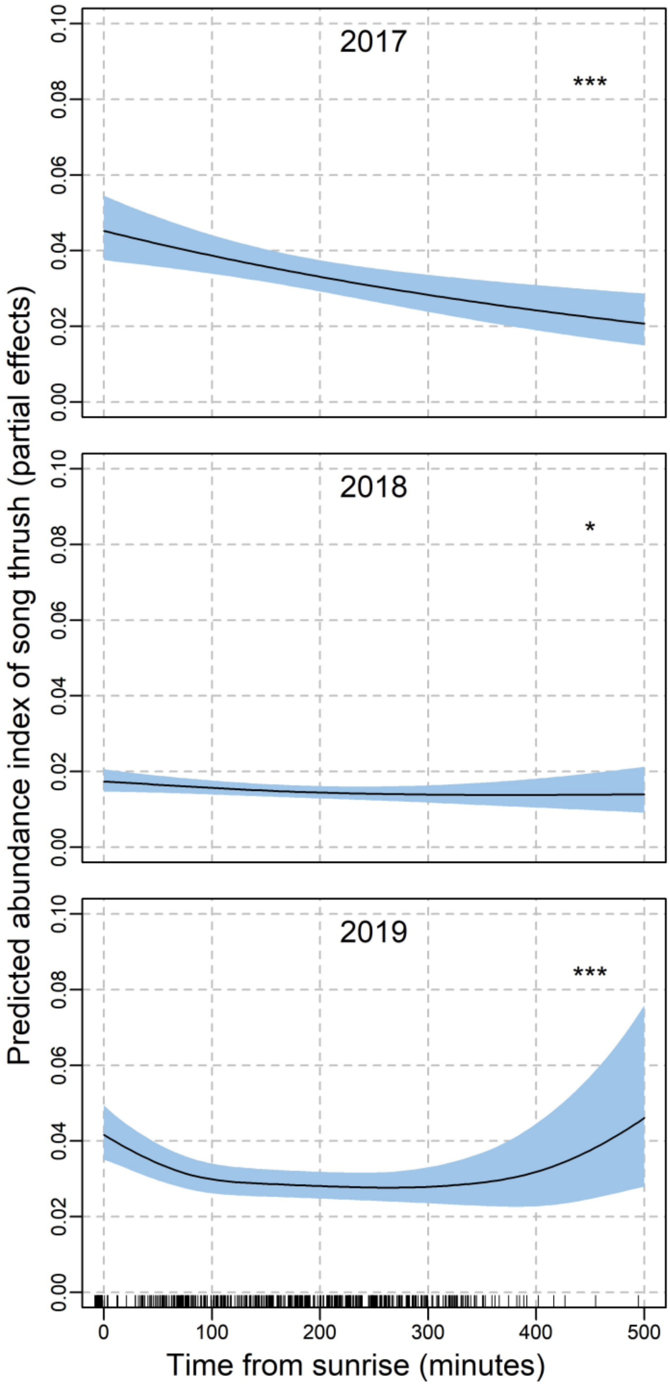

Figure S5. Effect of the starting survey time (minutes from sunrise) on the abundance index of song thrushes each year. Estimates from generalized additive mixed models with Poisson error distribution. In every model survey duration (in minutes) was included as the offset variable. Ribbons are 95 % confidence intervals. Explanations: * - $p < 0.05$, *** - $p < 0.001$.

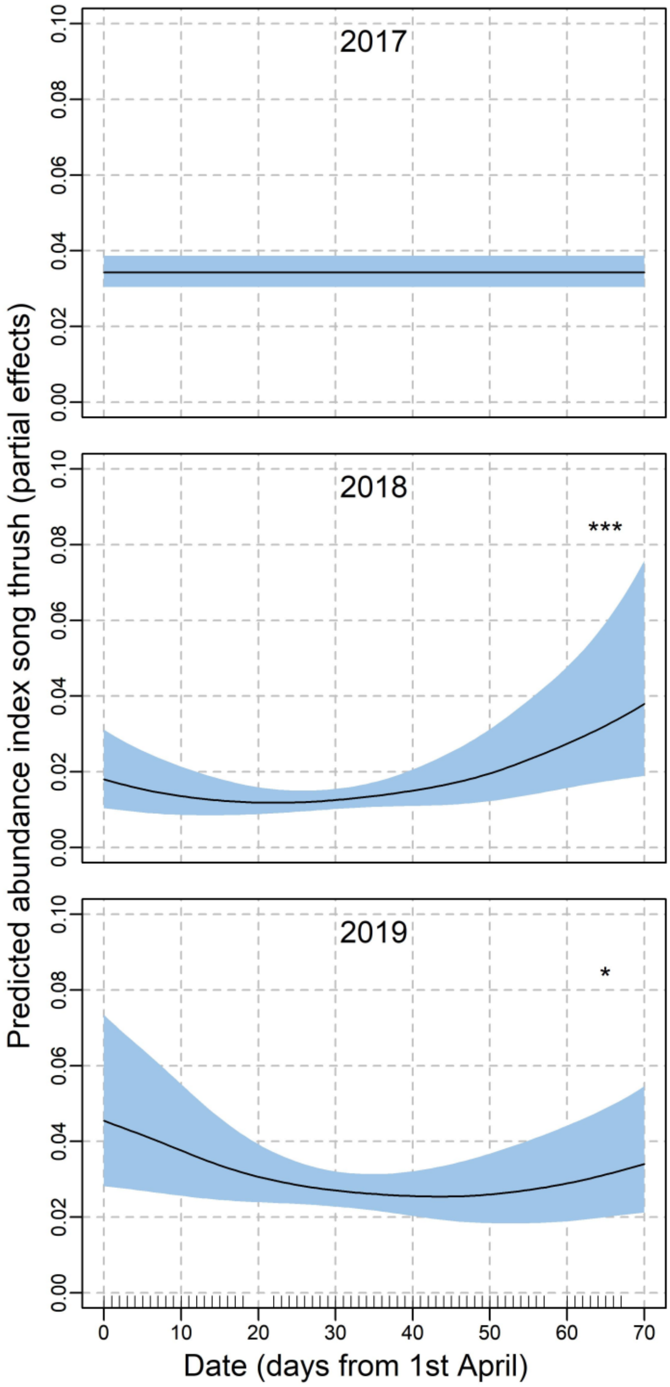

Figure S6. The effect of survey date (expressed as the number of days from 1st April) on the abundance index of song thrush. Estimates from generalized additive mixed models with Poisson error distribution. In every model survey duration (in minutes) was included as the offset variable. Ribbons are 95 % confidence intervals. Explanations: * - $p < 0.05$, *** - $p < 0.001$.

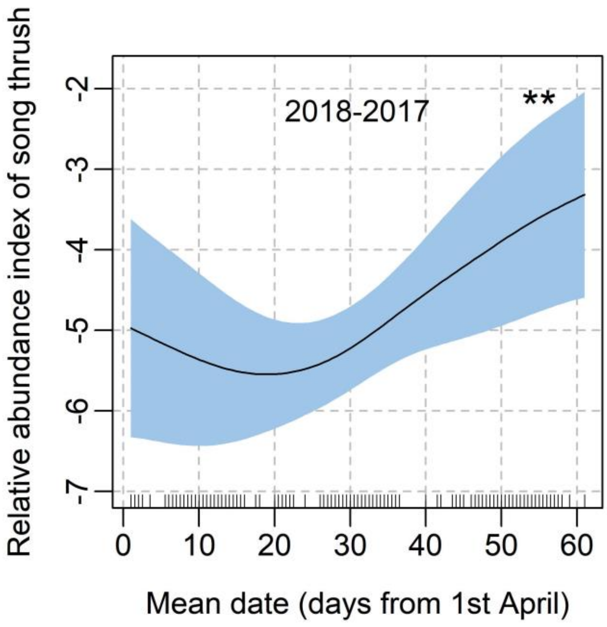

Figure S7. The effect of mean date (expressed as the number of days since 1st April) on the relative abundance index of song thrush (difference in abundance index between 2018 and 2017). Estimates from the generalized additive mixed model with Gaussian error distribution. Total survey duration (in minutes) was included as the offset variable. Ribbons are 95 % confidence intervals. Explanations: ** - $p < 0.01$.

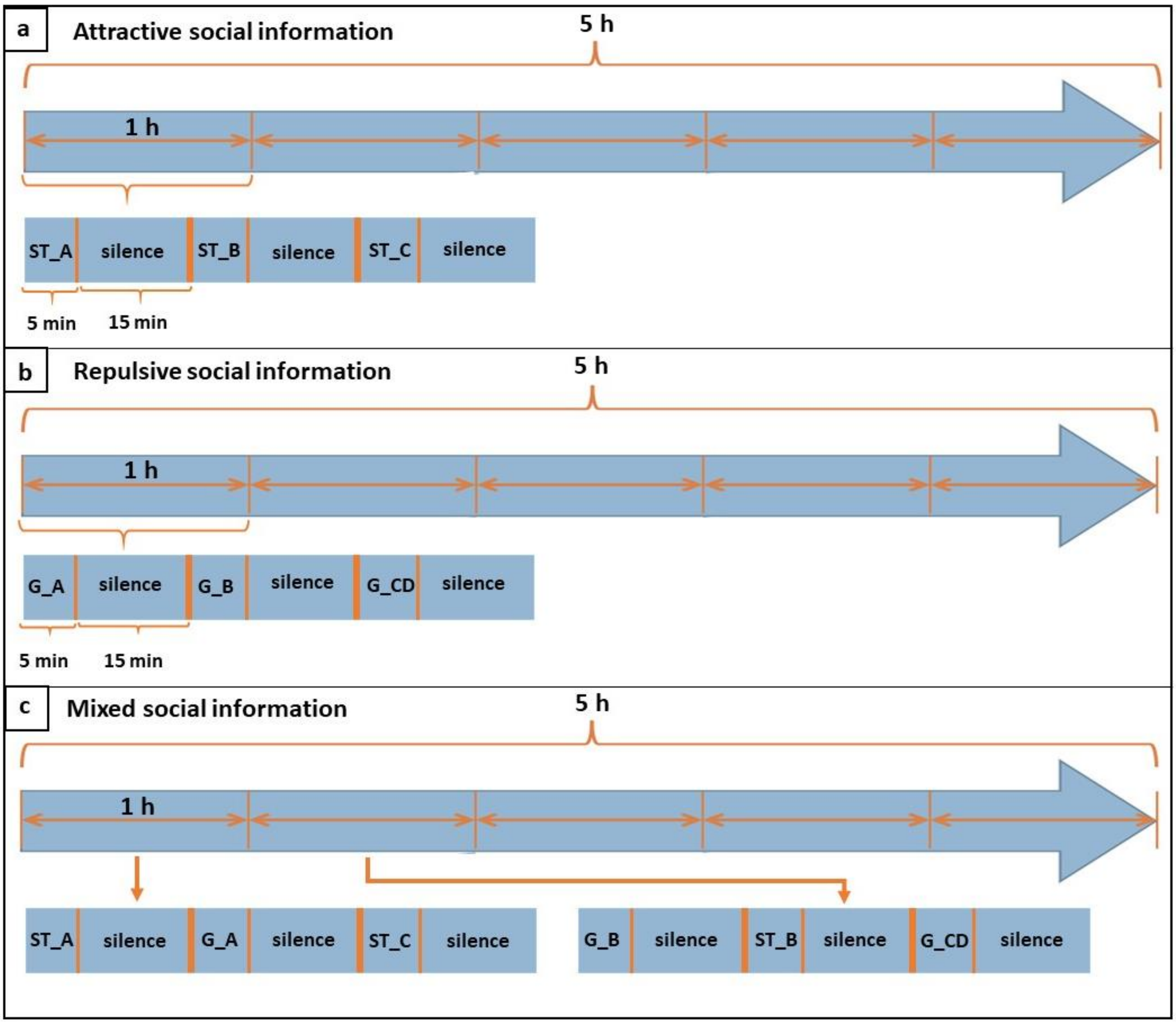

Figure S8. Scheme of the used broadcasts of the attractive (a), repulsive (b), and mixed (c) social information. Abbreviations used: ST – song thrush, G – goshawk, A, B, C, D – sound samples of particular individuals.

Table S1. The mean characteristics (area and isolation index) of the studied forest patches across the experimental groups. Forest area is in hectares and forest isolation index is the nearest-neighbour distance in meters. Explanations: SD - standard deviation, Min - minimal value, Max - maximal value.

	Area				Isolation			
Broadcast type	Mean	SD	Min	Max	Mean	SD	Min	Max
Control	14.3	13.91	0.4	57.5	610.8	666.96	35	2865
Procedural control	16.0	17.68	0.4	63.2	445.6	690.34	17	3295
Positive	12.8	16.68	0.4	56.3	761.4	890.24	17	3509
Repulsive	20.4	26.41	0.7	102.3	565.5	720.38	18	3295
Mixed	11.1	18.70	1.4	84.1	405.0	602.47	33	2469

Table S2. Test of differences in patch area among forest patches assigned to different treatments. Results of the general linear additive model with broadcast type as a factor and interaction between geographic coordinates as a smooth term. Explanation: CI - 95% confidence interval.

	**Area**		
Predictors	*Estimates*	*CI*	*p*
(Intercept)	14.55	7.62 – 21.47	**<0.001**
Broadcast type [Procedural control]	1.29	-8.53 – 11.10	0.796
Broadcast type [Attractive]	-1.16	-10.99 – 8.67	0.815
Broadcast type [Repulsive]	5.21	-4.62 – 15.04	0.296
Broadcast type [Mixed]	-3.51	-13.35 – 6.33	0.482
Smooth term (X,Y)			0.751

Table S3. Test of differences in patch isolation among forest patches assigned to different treatments. Results of the general linear additive model with broadcast type as a factor and interaction between geographic coordinates as a smooth term. Explanation: CI - 95% confidence interval.

	**Isolation**		
Predictors	*Estimates*	*CI*	*p*
(Intercept)	609.08	393.88 – 824.28	**<0.001**
Broadcast type [Procedural control]	-177.35	-483.63 – 128.94	0.254
Broadcast type [Attractive]	131.09	-173.23 – 435.41	0.395
Broadcast type [Repulsive]	-44.10	-351.84 – 263.63	0.777
Broadcast type [Mixed]	-166.70	-476.26 – 142.87	0.289
Smooth term (X,Y)			**<0.001**

Sound References:

Northern goshawk A

Piotr Szczypiński 2017. *Accipiter gentilis*, XC358782. www.xeno-canto.org/358782

Northern goshawk B

Dawid Jablonski 2014. *Accipiter gentilis*, XC165760. www.xeno-canto.org/165760

Northern goshawk C

Piotr Szczypiński 2017. *Accipiter gentilis*, XC358611. www.xeno-canto.org/358611

Northern goshawk D

Piotr Szczypiński 2017. *Accipiter gentilis*, XC358781. www.xeno-canto.org/358781

Song thrush A

Antoni Knychała 2017, *Turdus philomelos*, XC367050. www.xeno-canto.org/367050

Song thrush B

Antoni Knychała 2013, *Turdus philomelos*, XC138645. www.xeno-canto.org/138645

Song thrush C

Joachim Rupik 2012, *Turdus philomelos*, XC105123. www.xeno-canto.org/105123
